# Geodynamic subduction models constrained by deep earthquakes beneath the Japan Sea and eastern China

**DOI:** 10.1038/s41598-020-62238-x

**Published:** 2020-03-25

**Authors:** Hana Čížková, Jiří Zahradník, Junqing Liu, Craig R. Bina

**Affiliations:** 1 Charles University, Faculty of Mathematics and Physics, Department of Geophysics, Prague, Czech Republic; 2 Jilin Earthquake Agency, China Earthquake Administration, Changchun, China; 3 Northwestern University, Department of Earth and Planetary Sciences, Evanston, IL USA

**Keywords:** Solid Earth sciences, Geodynamics

## Abstract

Details of Pacific plate subduction under the Japan Sea and associated current seismicity remain challenging. Seismic tomography reveals a continuous slab dipping at ~30° down to ~600 km, and earthquake mechanisms point to down-dip compression. Further, the slab is lying at the 660-km discontinuity, and this zone is aseismic. We suggest that this pattern results from the slab’s negative thermal buoyancy, resistance of the viscous lower mantle, and buoyancy forces associated with the phase transitions at 410 km and 660 km. Our model comprises an ageing subducting plate, nonlinear rheology and major phase transitions. The model explains the observed low dip angle of the slab and predicts a detailed stress pattern related to bending down to 450 km, followed by unbending as the slab is laid flat upon the 660 km boundary. Remarkably, in the bending/unbending regions, down-dip compression occurs close to the slab top/bottom, respectively. As only down-dip compression is observed, we argue that the earthquakes are mapping the top and bottom of the slab. The absence of seismicity in the flat-lying slab is explained by significantly lower stresses and higher temperatures. With this new knowledge, increasingly accurate seismic locations will considerably improve images of finite-extent slab geometry.

## Introduction

Present knowledge of viscoelastic structure and mineralogy provides advanced models of lithospheric slabs and their subduction in the mantle^1^. When modeling a specific region of the world, several competing models can be constructed. Their common features are determined by the key model assumptions and parameters, such as rheology or thermomechanical properties of mantle material. However, due to variations of (and uncertainties in) many others – such as slab age, properties of the overriding plate, and properties of the decoupling layer between the subducting and overriding plates – such models result in large variety of slab morphologies and thermal structures. In order to tailor them to specific natural subduction regions, subduction models should be constrained by independent seismic observations, e.g. tomography, seismicity, focal mechanisms, principal stress axes. We call these geodynamic-seismic models. Though there are numerous subduction modeling studies that investigate stress evolution in kinematic models with predefined subduction geometry, thermal structure and/or velocity^2–6^, there are only a few that study stress evolution in a fully dynamic setup^7–11^ and relate it to the stress derived from seismicity in a natural subduction area^12,13^. Particularly interesting are physical conditions at the deepest slab ‘tips’, determining interaction with the 660-km discontinuity, e.g. temporary stagnation of slabs. Searches for suitable geodynamic-seismic models are challenging because deep-focus earthquakes are rare and their physical nature is still puzzling^14^.

The aim of this paper is to provide a geodynamic-seismic model for the subduction of the Pacific plate beneath the Japan Sea and Eastern China. In this region, according to tomography investigations^15–19^, the subducting slab stagnates on the 660 km discontinuity. Seismicity is almost continuous from 0 to 600 km. The deepest activity (600 km) terminates at longitude 130° E, while the horizontal part of the slab continues at least some 800 km towards the west in aseismic mode. We present a suite of possible models that should predict the seismological observations: slab morphology, seismicity and stress orientations. The goal is not to perfectly fit the observed slab (which would hardly be possible in a simplified 2D free-subduction model) but rather to decipher the controls on slab deformation and stress state. As a byproduct, we clarify certain physical conditions at which deep-focus earthquakes may occur in the region, characterized by the second stress invariant and temperature.

## Pacific Plate Beneath Northeast China

In northeast China, the Pacific plate is subducting beneath the Eurasian plate. The subduction rate at the Japan trench is 9–10 cm/yr. Slab thickness is about ~90 km^20^. In tomographic models (e.g.^15,16,18^), many of which are accessible through SubMachine Web-Based Tools^21^, the plate is clearly represented by a high-velocity anomaly extending from the surface to stagnation at ~600 km. Inside the plate, a continuous Wadati-Benioff zone can be traced^22^. A fast seismic anomaly horizontally lying above the 660-km interface continues further towards the west and terminates (seismic velocity anomaly reaching 0) about 800–1400 km from the point where the slab reaches 660 km depth.

Slow velocity anomalies have been detected above the stagnant slab, giving rise to speculations about the origin of active intraplate volcanoes, e.g. Changbai, via asthenospheric injection^23^ or via deep slab dehydration and convective circulation in the mantle wedge^19,20^. At the greatest depths of the slab beneath northeastern China, a metastable olivine wedge has been proposed^24^, characterized by locally decreased P-velocity and density, possibly inducing positive buoyancy^4^. Down-dip compression was indicated by numerical models (e.g.^2^) and by focal mechanisms^25^. The recently emerging question of whether the interiors of deep slabs are strongly anisotropic is also challenging^26,27^ because it might imply presence of magnesite or carbonatite melt, potentially important for the generation of deep-focus events.

Kinematically driven models of the region^6,28^ are consistent with major effects such as slab stagnation with a low dip angle. Recently, the paper^13^ presented a model of the geodynamic evolution of northeast Asia and demonstrated that in a setup tailored for the region, namely the changing age of the subducting plate and predefined weakness zones in the overriding plate, they are able to explain the timing of extensional episodes. They briefly discussed also the stresses in the slab; their model indicates along-slab compression below 200 km depth.

**Figure 1:**
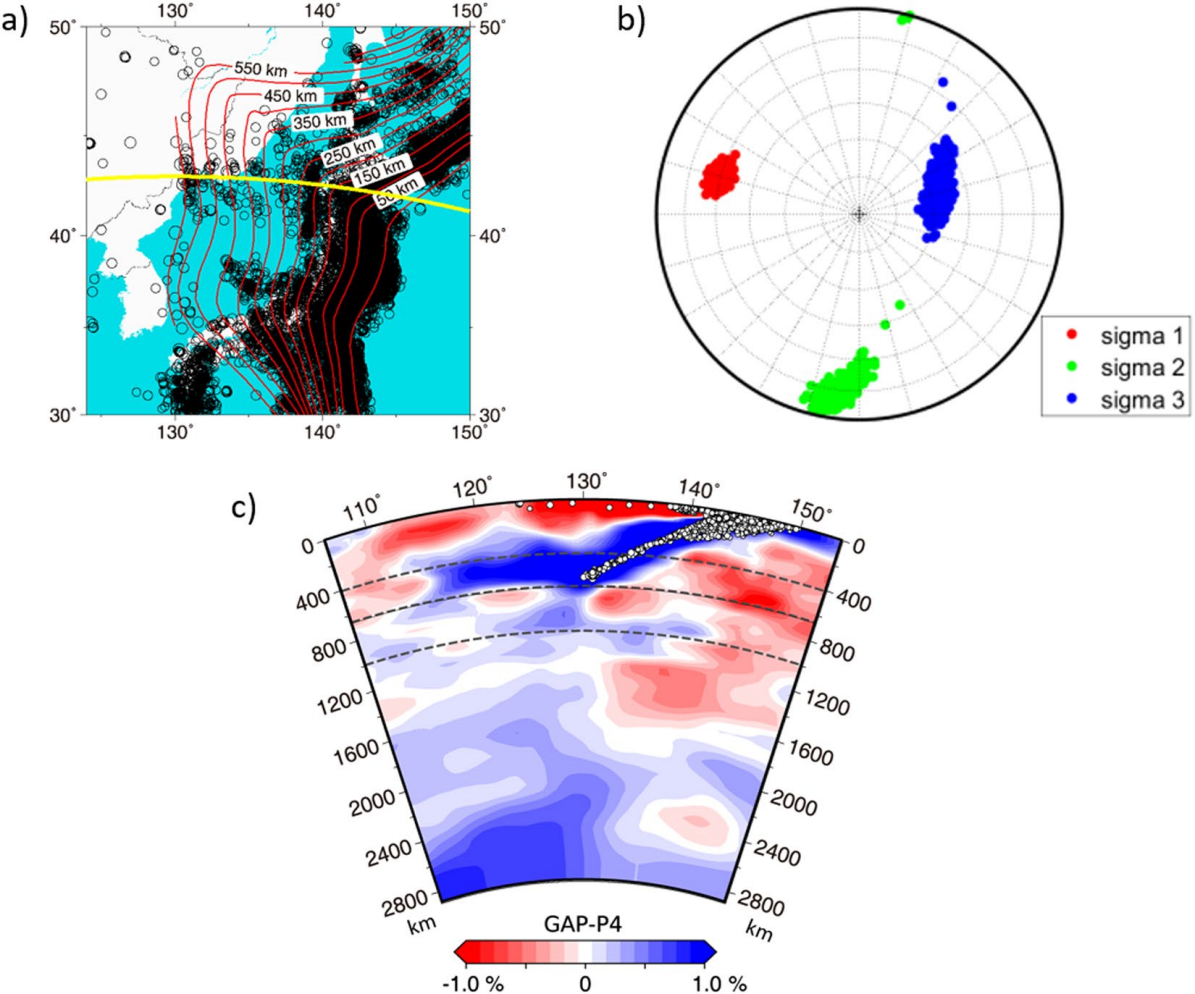
Seismicity, stress and tomography. (**a**) Earthquake epicenters in the study region between 1960 and 2014, magnitude >3.3. Isolines of the slab depth according to model RUM. Yellow line represents a part of the great circle arc centered at latitude 43°, longitude 130°, azimuth 90°. (**b**) Principal stress axes derived from focal mechanisms at depths 300–600 km. (**c**) Hypocenters (white circles) superimposed with the tomographic model GAP-P4. The cross-section follows the arc partly shown by the yellow line in panel (**a**) and extends ~20° further to the west. Projected on the cross-section are earthquakes situated +/−200 km off the yellow-line profile. Map in panel (a) was plotted with GMT-4.5.18 (https://www.generic-mapping-tools.org). Tomographic image in panel (**c**) was plotted with a SubMachine web tool (https://www.earth.ox.ac.uk/~smachine/cgi/index.php).

One of the most interesting features of the studied region is that although earthquakes occur down to 600 km, a significant part of the stagnant slab (longitude 115°–130°) under northeast China is aseismic. Similar behavior can be observed at other slabs of the world (e.g., IZU, KUR, TON in Fig. 1 of the paper^1^). Here we aim at explaining the observed seismogenic stresses in terms of the dynamic effects of the petrological buoyancy associated with major phase transitions and viscous resistance of the stiff lower mantle.

### Seismology constraints

According to catalogs of the International Seismological Center (ISC, before 2015) and the United States Geodetic Survey (USGC, 2015 and later), seismicity below 300 km forms a relatively narrow (less than 50-km thick), almost planar Wadati-Benioff zone dipping at ~30° and extending down to 600 km (Fig. 1a). Here, isolines of the slab depth according to model RUM are included^29^. Previous studies of focal mechanisms, mostly based on the Global Centroid Moment Tensor project (GCMT), have indicated that the P-axes of earthquakes deeper than 300 km are basically aligned with the Wadati-Benioff zone (e.g.^25^, their Fig. 15). Using the GCMT mechanisms of 39 events from the study region (depth 300 to 600 km, confined in a band +/−200 km from the profile marked by a yellow line in Fig. 1a, Mw 5.0 to 6.9) we confirmed this predominant P-axis orientation.

**Figure 2:**
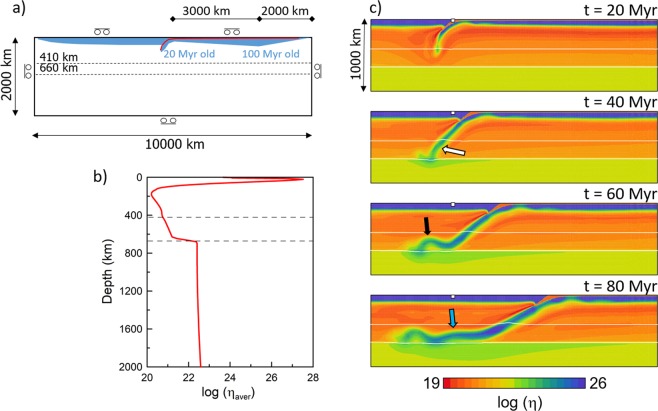
(**a**) Model setup. Cartesian domain is 2000 km deep and 10000 km wide. Subducting plate on the right is young (20 Myr) at the trench then its age increases for 3000 km and reaches maxima (100 Myr) 2000 km from the ridge. Red line marks the weak decoupling layer. Dashed lines indicate the major mantle phase transitions. (**b**) Horizontally averaged viscosity in the model domain. Dashed lines indicate the positions of the phase transitions included in the study. (**c**) Four snapshots of the time evolution of viscosity in model M1. Part of the model domain 1000 km deep and 4000 km wide is shown. White lines indicate the positions of the phase transitions at 410 km and 660 km depths; white squares show an initial position of the trench. Arrows mark the weakening of the slab in the transition zone (white arrow) and forming of the first and second buckle (black and blue arrows).

To quantify the stress field, we inverted the GCMT focal mechanisms for directions of principal stress axes (Fig. 1b) using the StressInverse method^30^. The compressional axis σ1 is well resolved, characterized by azimuth/plunge = 284°/27°, whose uncertainties are ~ +/−10°. This result is almost identical to that of the paper^31^, their Fig. 6. Considering separately 12 events in the shallower range of 300–450 km and the same number of events in the deepest range 550–600 km, we obtained slightly different σ1 azimuth/plunge values = 292°/35° and 272°/22°, respectively. It appears that the plunge decreases with increasing depth, i.e., the σ1 axis becomes less steep. However, this apparent decrease in plunge falls just within the range of associated uncertainties.

Tomography investigations show that the Wadati-Benioff zone is embedded in a high-velocity subducting slab, that reaches 660 km depth at longitude ~130° and terminates at ~115°. The zone between longitude 115° and 130°, where the slab is stagnant, is clearly aseismic. These features illustrated in Fig. 1c on a cross-section taken from model GAP-P4^16^ are stable across several tomography models (e.g.^17,18^).

In order to explain the above observations, we construct a numerical model of the long-term tectonic evolution of the area. According to the paper^32^, subduction of the Pacific plate under eastern China started some 60 Myr ago by subducting the Izanagi-Pacific ridge. We will therefore assume that initially very young lithosphere at the trench is followed by rapidly ageing plate^13^. Our free-subduction model then evolves without kinematic presumptions, and slab behavior results from the combination of internal driving forces – thermal and compositional buoyancy and petrological buoyancy associated with the major phase transitions. We aim at predicting the dip angle of the slab, stress orientations in the slab, and the aseismic nature of the flat-lying portion that extends ~1000 km towards the west from the point where the slab first encounters the 660 km interface.

### Subduction model setup

In order to capture the key features of slab deformation under conditions typical for the western edge of the Pacific plate, we apply a 2D Cartesian model of subduction in a model domain 2000 km deep and 10000 km wide. We use an extended Boussinesq approximation of basic equations and apply the finite element method as implemented in the SEPRAN package^33^. The subducting Pacific plate initially stretches from the ridge in the upper right corner of the model domain to the trench located in the middle of the upper surface (Fig. 2a). An overriding plate lies between the trench and another ridge positioned in the upper left corner of the model box. The subducting plate is covered by a 10-km thick weak oceanic crust-like layer that effectively decouples the plates. Its low viscosity supresses friction at the contact of the plates and facilitates subduction. In some models this crust is further assumed to be more buoyant than underlying lithosphere, with the density contrast of the oceanic crust being −200 kg/m^3^.

The initial condition for temperature distribution in the model domain should be representative of East Pacific in early Cenozoic. The thermal structure of the overriding plate follows a halfspace cooling model and reaches the age of 100 Myr at the trench. The initial thermal structure of the subducting plate follows^13^: the age of the subducting plate at the trench is 20 Myr, while from there to the right it ages rapidly to reach the age of 100 Myr at a distance of 3000 km from the trench, and then the age decreases towards the ridge following the halfspace model (Fig. 2a). Temperature distribution below the plates follows an adiabat with a potential temperature of 1573 K. Thermal expansivity decreases with depth from a surface value of 3 × 10^−5^ K^−1^ to 1.2 × 10^−5^ K^−1^ at 2000 km depth (for details see Supplementary Information). Constant temperature is prescribed as the surface boundary condition (273 K) as well as at the bottom boundary (2130 K). Side boundaries are reflective. A short kinematic prerun with a prescribed constant velocity on the top of the subducting plate is first executed. During this 7-Myr long run a tip of the subducting plate is driven to a depth of 200 km. Then the driving velocity is switched off and the subsequent model runs are driven purely by the slab buoyancy, with impermeable free-slip prescribed on all boundaries.

The deformation of the lithosphere and upper mantle material is controlled by a composite rheological model that combines diffusion creep, dislocation creep and a power-law stress limiter^34^. In the lower mantle we assume diffusion creep with parameters derived from the analysis of slab sinking speed^35^. Figure 1b shows the horizontally averaged viscosity profile. The decoupling crust has viscosity of 10^20^ Pas. Details of the rheological model as well as model parameters are given in the Supplementary Information.

Major mantle phase transitions are included in the model – the olivine-wadsleyite transition at 410 km depth and the transition of ringwoodite to bridgmanite and ferropericlase at 660 km depth. Since this simplification of the mantle composition to the olivine component is rather restrictive^36^ we test the sensitivity of the system to the strength of these transitions. We thus use two combinations of Clapeyron slopes: in one set of models (referred to as ‘weak phase transitions’) we assume that γ_410_ = 1 MPa/K and γ_660_ = −1.5 MPa/K, while the other set (‘strong phase transitions’) has γ_410_ = 2 MPa/K and γ_660_ = −2.5 MPa/K. Both buoyancy and latent heat effects associated with the phase transitions are taken into account.

### Geodynamic model of subducting pacific slab

Here we summarise the results of several model runs that attempt to explain the evolution of the Pacific slab under the Japan Sea. We are particularly interested in several characteristic features: 1) the morphology of the subducted slab with a low dip angle of ~30°, 2) the flat-lying portion of slab resting at the boundary at 660 km depth and extending several hundred kilometers towards the west beyond the point where the slab first reached the 660-km discontinuity, and finally 3) the stress distribution that is in agreement with mechanisms of deep earthquakes.

In Fig. 2 we illustrate the time evolution of the reference model M1 that has weak and buoyant crust and assumes weaker phase transitions. We show four snapshots of viscosity in a part of the model domain 1000 km deep and 4000 km wide. In the initial stage of the subduction process, a young lithosphere (~20 Myr old) is subducting at a steep dip angle and its limited negative buoyancy results in rather slow subduction velocities (less than 1 cm/yr in the first 20 Myr). The accelerating effect of an exothermic phase transition at 410 km depth, together with the fact that successively older plate arrives at the trench, results in an increasing plate velocity (maximum 4 cm/yr at ~22 Myr). As the slab tip arrives at the bottom of the transition zone, the resistive buoyant force of the endothermic phase change combined with the resistance of a more viscous lower mantle initiates trench rollback and associated decrease of the dip angle (Fig. 1c, 40 Myr). The slab is rolling back, the dip angle in the upper 400 km is decreasing, and a flat-lying part of the slab develops above the phase boundary at 660 km depth (Fig. 2c, 60–80 Myr).

**Figure 3:**
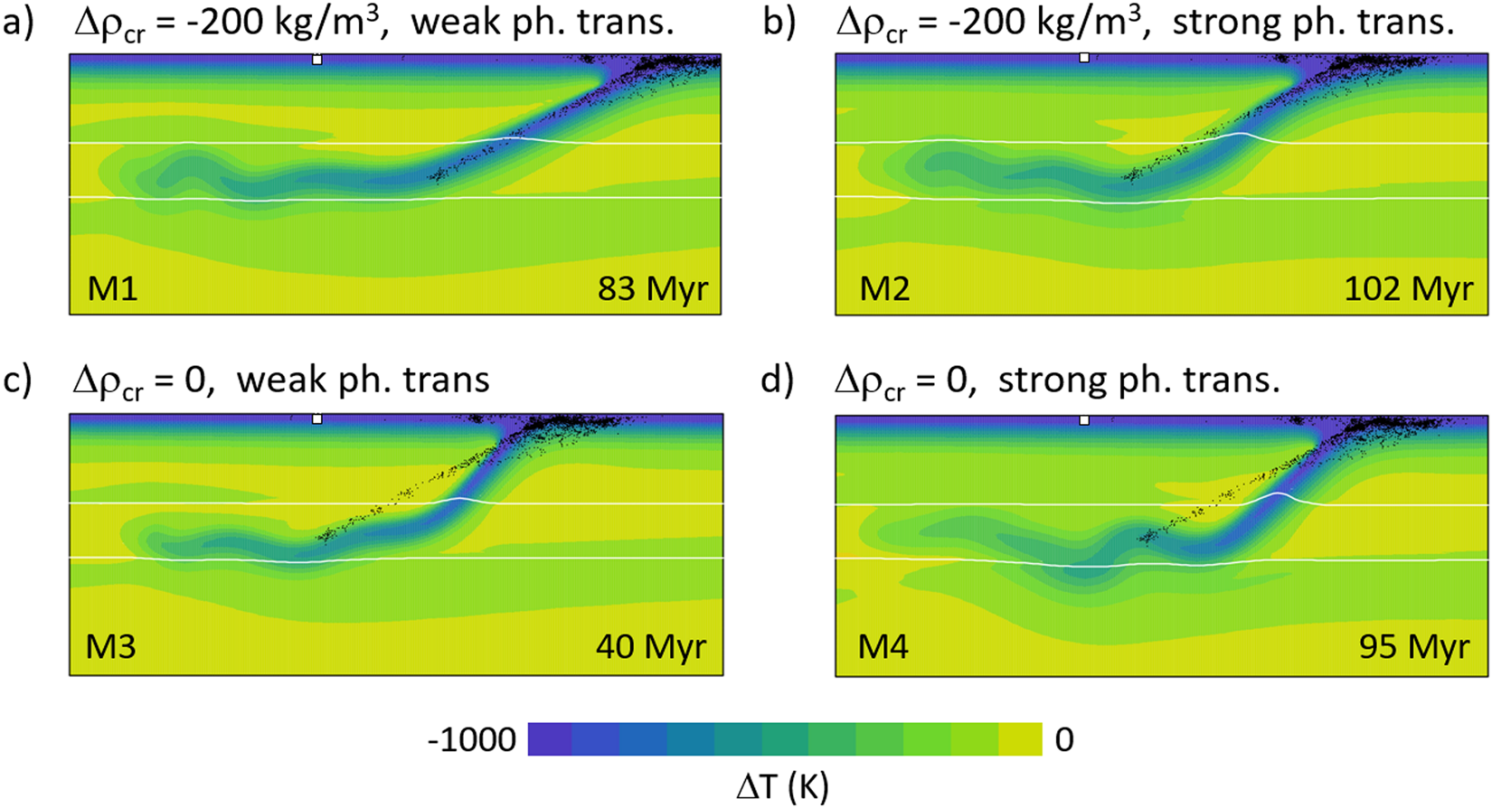
One snapshot of temperature anomaly (with respect to the adiabatic profile). Part of the model domain 1200 km deep and 3000 km wide is shown. White lines indicate the positions of the phase transitions at 410 km and 660 km; white square marks the initial position of the trench. Black dots show the same earthquakes as in Fig. 1a. (**a**) Model M1 with buoyant crust and weak phase transitions, snapshot taken at 83 Myr. (**b**) Model M2 with buoyant crust and strong phase transitions, snapshot taken at 102 Myr. (**c**) Model M3 with weak phase transitions, snapshot taken at 40 Myr. (**d**) Model M4 with strong phase transitions, snapshot taken at 95 Myr.

**Figure 4:**
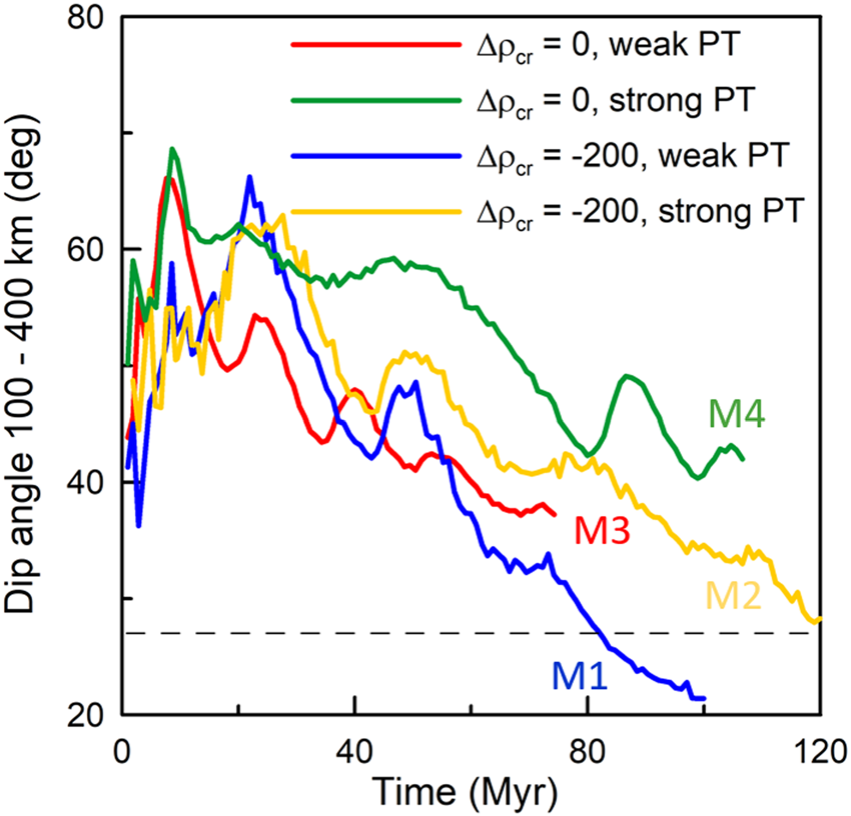
Dip angle as a function of time for models M1–M4. Model dip angle is measured in the depth interval 100–400 km using markers placed at the top layer of the slab. Dashed line indicates slab dip angle as inferred from the locations of the earthquakes shown in Fig. 3. Model curves are shown until the moment when the flat lying part of the slab extended 1500 km and the calculation was terminated.

In Fig. 3a one snapshot of this model M1 is shown. We plot here the temperature anomaly (with respect to the mantle adiabat) with the earthquake foci overprinted. The snapshot is taken at 83 Myr, the moment when the slab geometry is roughly in agreement with the tomographic indication (cf. Fig. 1c). Clearly the dip angle of the model slab agrees well with the dip of the Wadati-Benioff zone (27°). Since Fig. 3a represents only one moment in the model evolution, let us check the correspondence of the slab shape with the dip angle of the Wadati-Benioff zone over a longer period of time. To that end we evaluated the time evolution of the model slab dip angle (measured in the depth interval 100–400 km) and compared it with the observed slope of the Wadati-Benioff zone. Model dip angles are illustrated in Fig. 4, where the blue line is for model M1 discussed above. Initially, the dip angle is increasing as the slab tip starts sinking into the upper mantle and reaches the 410-km discontinuity. A maximum dip angle of 65° is attained at ~20 Myr, then it is decreasing while trench rollback is initiated as the slab descent is hindered by the resistance of the 660 km discontinuity. The trench then retreats and the dip angle decreases until ~40 Myr. At that time the slab material in the transition zone is weakened due to the combined effects of the petrological buoyancy forces associated with the phase transitions and ongoing rollback (see white arrow in Fig. 2c, t = 40 Myr), and a horizontal fold forms (black arrow in Fig. 2c, t = 60 Myr). During this buckling period shallow portions of the slab rotate towards vertical, and the dip angle increases locally. After the fold is finished (~50 Myr), the dip angle decreases again. A similar (though much less pronounced) local maximum is observed at ~72 Myr when a weaker second buckle (blue arrow in Fig. 2c, t = 80 Myr) is forming. Thus, in the time interval 70–80 Myr the model dip angle is close to the observed value of 27°. After 80 Myr, the dip angle is further decreasing while the slab is rolling back. The flat-lying part of the slab remains stable in the transition zone, but its extent grows to exceed that of the anomaly as indicated by seismic tomography.

If we assume stronger phase transitions and weak buoyant crust (model M2), slab morphology is similar, though with some differences. In Fig. 3b we show a snapshot taken at 102 Myr. At this time the slab has evolved into a position similar to model M1 at 83 Myr (cf. Fig. 3a,b). The rollback is reduced in model M2 with respect to model M1, due to a stronger Clapeyron slope of the 410-km phase transition (Čížková & Bina, 2013), and thus the slab morphology resembles that of model M1 at a much earlier moment. The main difference between the slabs in Fig. 3a,b is in dip angle – model M2 with stronger phase transitions results in a slab steeper than the observed seismicity. If we compare the time evolution of the dip angle in these two models (cf. blue and yellow lines in Fig. 4), we observe that with stronger phase transitions the dip angle evolves similarly as with weaker phase transitions, only the reduction of the dip angle towards the observed 27° takes considerably longer. This low dip angle is obtained only after 110–120 Myr, and at that time the flat-lying portion of the slab at 660 km is already too long (more than 1500 km).

The above two models M1 and M2 both possessed a buoyant crust. Now we demonstrate the behavior of the corresponding models that still have weak crust but are not associated with extra (compositional) buoyancy. Clearly both model M3 with weak phase transitions and model M4 with stronger phase transitions (Fig. 3c,d) are dipping more steeply than the corresponding models M1 and M2. Since the crust is not compositionally buoyant, slabs in models M3 and M4 clearly miss an important contribution to slab unbending at shallow depths. The slab dip evolution with time (Fig. 4, red and green lines) shows that the dip angle in these two models remains higher than ~37°.

**Figure 5:**
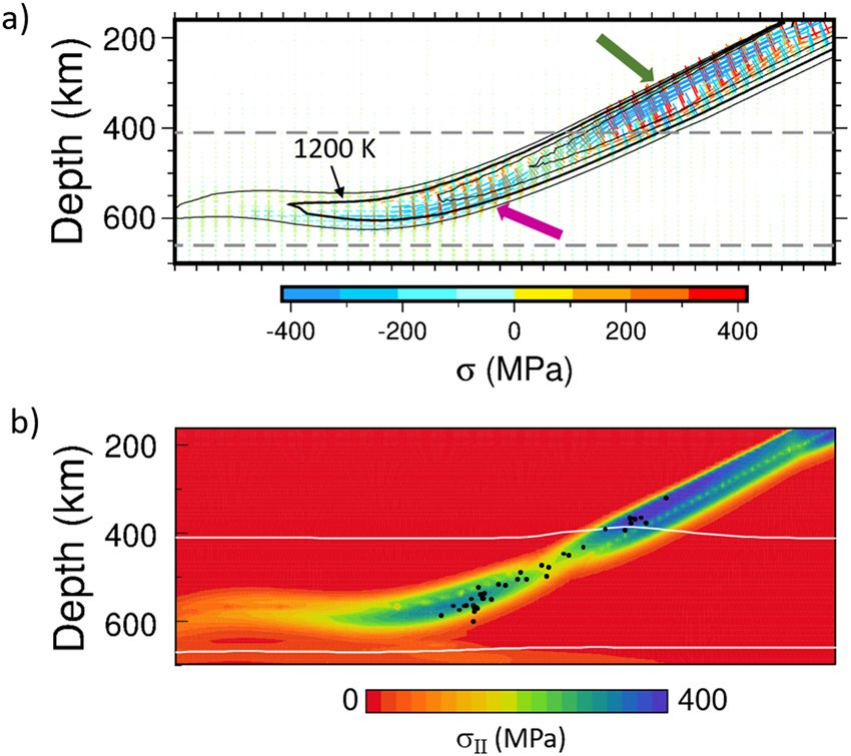
Deviatoric stress in a zoomed area around the slab in model M1 at 83 Myr. (**a**) Principal stress axes. Blue is for compression, red for extension. The length of the bars corresponds to the principal stress amplitude which is also indicated by color. Overprinted are the isolines of temperature; thick line corresponds to 1200 K and spacing between isotherms is 100 K. Green and purple arrows mark the parts of the slab subject to down-dip compression. (**b**) Second invariant of the deviatoric stress. Black dots show the locations of the deep events used in our stress analysis from focal mechanisms (depth 300 to 600 km, M_w_ 5.0 to 6.9). White lines indicate the positions of the major phase transitions. Stress plot in panel (**a**) was plotted with GMT-5.3.0. (https://www.generic-mapping-tools.org).

Based on the above considerations about slab dip angle and its fit to the observed geometry of the Wadati-Benioff zone, we selected the model M1 as the best fitting, and we further look more closely at the stress distribution in this model slab. Figure 5a shows the principal deviatoric stresses in a zoomed area of the slab. Blue indicates compression, red is for extension, and the color and size of the bars indicates stress magnitude. At shallower depths (200–400 km) the upper part of the slab is under down-dip compression while (a narrower) lower portion is experiencing down-dip extension. The deeper part of the slab (500–660 km) shows the opposite stress pattern, with down-dip compression now in the lower portion. Such a stress pattern is typical for bending and unbending – the subducting slab is divided into two layers of high stress, separated by a narrow ‘neutral’ plane characterised by low stresses that separates the compressing and extending layers during the bending/unbending process. The upper portion of the shallow part of the slab (marked by a green arrow in Fig. 5a) is compressed as the slab is bending while rolling back. The deeper part of the slab (purple arrow in Fig. 5a) is unbending as the slab is laid upon the boundary at 660 km, which results in down-dip compression in the lower portion of the slab. Moreover, the flat part of the slab is still slowly moving towards the west. The resistance of a more viscous lower mantle thus contributes to the horizontal compression in the slab at the 660-km boundary. Figure 5b illustrates the magnitude of the stress. It shows the second invariant of the deviatoric stress tensor σ, i.e., [0.5 Σσ_ij_σ_ij_]^1/2^. Clearly, the upper layer of the slab above and around 410 km shows higher stress magnitudes than the lower slab layer. Deeper in the transition zone it is the opposite: the higher stress is obtained in the lower portion of the slab. The foci of the 39 deep earthquakes M_w_ >5 that were used in our seismic stress analysis are printed over the stress-magnitude plot. If we take into account the fact that only down-dip compressional earthquakes are observed in the depth interval 300–600 km, the positions of the foci may suggest that the earthquakes occur in the more stressed upper layer of the slab above and around 410 km depth while in the deep transition zone they occur in the lower slab portion (down-dip compressed in both cases). It appears that stress magnitude is gradually decreasing as the slab warms with increasing depth (Fig. 5a).

**Figure 6:**
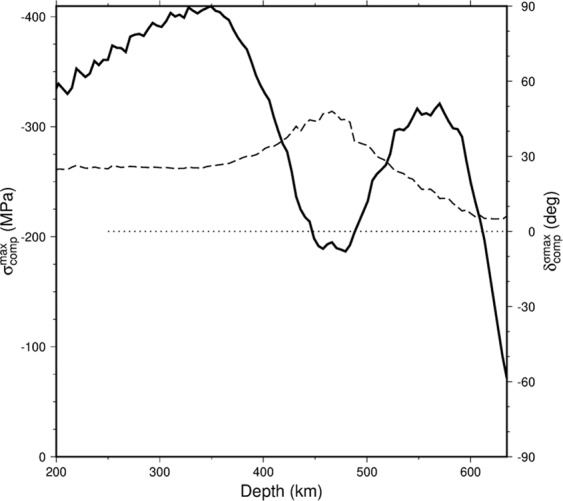
Solid line shows the amplitude of maximum compressional stress *σ*^max^_comp_ as a function of depth. Dashed line is for the dip angle (plunge) of the maximum compressional stress δ^*σ*max^_comp_.

Finally, Fig. 6 shows the amplitude of the maximum compressional stress σ_max_ as a function of depth (solid line) together with its dip angle (dashed line). In the upper mantle a relatively high stress magnitude (~400 MPa) is observed associated with slab bending during rollback. In the transition zone another local stress maximum (~300 MPa) corresponds to unbending of the slab as it is laid upon the phase boundary. These two maxima are separated by a minimum at ~450 km depth where the stress regime changes from bending to unbending. At the same depth there also appears to be a local decrease in the density of seismicity. The dip of the maximum compressional axis is fairly uniform in the upper mantle and is generally oriented in the down-dip direction. Then it increases locally while the stress orientations change, and finally it decreases continuously across the transition zone and approaches the horizontal (δ^σmax^ = 0) at the base of the transition zone, where slab stagnation occurs.

## Discussion and conclusions

We have demonstrated that free-subduction models predict slab morphology consistent with tomographic indications in the Japan Sea – Eastern China region if the initial thermal structure of the subducting plate corresponds to that of the young plate at the trench followed by rapidly ageing lithosphere^13,32^. A young initial slab tip turns out to be a crucial feature. We have also tested models in which the initial thermal structure of the subducting plate followed a halfspace model reaching the age of 100 Myr or 150 Myr at the trench. In these models the developed slab geometry would not fit the observed Pacific slab morphology regardless of the strength of the phase transitions. Such models with an old, cold and thus heavy plate at the trench inevitably result in the development of a backward deflected tip at the 660-km boundary, and in cases of weaker phase transitions this tip even penetrates into the shallow lower mantle.

Phase transitions constitute crucial factors that affect the deformation of slabs at the base of the upper mantle (e.g.^4,38–40^). Stresses generated by petrological buoyancy due to phase transitions in the slab yield viscosity reduction in the nonlinear rheological model and result in slab buckling in the transition zone. Strong phase transitions imply more buckling and reduced trench rollback, while weaker phase transitions result in faster trench rollback^37^. Here we show that faster trench rollback associated with weaker phase transitions yields also lower dip angles, and therefore the models with weak phase transitions fit the observed low slab dip angle more successfully. The Clapeyron slopes of our weak phase transition models (γ_410_ = 1 MPa/K, γ_660_ = −1.5 MPa/K) are at the lower end of the values that are commonly used in geodynamical modelling. For an exothermic transition at 410 km, values as high as 3 MPa/K were reported, and an endothermic phase change at 660 km may have a Clapeyron slope of −2.5 to −4 MPa/K assuming purely olivine mantle composition^41–44^. The pyrolitic mantle has, however, a more complex phase transition diagram, and it has been shown that assuming purely olivine composition overestimates the effects of the phase changes on slab deformation and exaggerates the folding and stagnation^36^. Our weaker phase transition models (M1 and M3) that were shown to produce a better fit to slab dip angle (compared to strong phase transition models) may thus turn out to be the more realistic ones.

The analysis of seismogenic stress associated with deep earthquakes (300–600 km) reveals down-dip compression characterized by azimuth/plunge = 284°/27° in the subducting slab. The stress pattern developed in our model slab in the preferred case with weak phase transitions and buoyant crust is characterised by down-dip compression in the upper layer of the slab at shallower depths (200–450 km) and in the lower portion of the slab in the deeper part of the transition zone. This stress pattern is typical for bending and unbending of the plate^7^. The magnitude of stress in these two parts of the slab is higher than in the complementary domains characteristic of down-dip extension. We therefore argue that the observed seismicity is confined to the domains of down-dip compressive stresses, while no strong events (M_w_ >5 used here) occurred in the complementary portions of the slab. The dip angle of the modeled main compressional axis is uniform (26°) up to ~400 km depth, then increases locally as the slab is turning from bending to unbending pattern, and further decreases towards the bottom of the transition zone, as is perhaps weakly indicated by seismic observations.

In the context of our model, observed seismicity appears to be largely confined to within the 1150 K isotherm, which may have implications for the feasibility of thermally sensitive potential seismogenic mechanisms such as transformational (metastable) faulting, adiabatic (shear) instability, or dehydration-mediated (embrittlement) rupture^14^.

While our model plate and rollback velocities do not individually match current regional estimates (rollback is faster while plate is slower), such values of “absolute” velocities are significantly dependent upon choice of reference frame^45^. Moreover, any such velocities relative to a mantle reference must also reflect global 3D flow fields which we cannot hope to capture in a regional 2D model. However, it is reassuring that the sum of our model plate and trench velocities^46,47^ is of the same order (~8 cm/yr) as estimated convergence rates.

The preferred model that best fits the observed slab morphology is associated with a compositional density anomaly – crust is more buoyant than the underlying lithosphere. We assume a relatively mild density contrast of 200 kg/m^3^, which is at the lower end of estimates of crust-mantle density contrast based on 3D gravity modelling^48^, ocean drilling^49^, and petrological modelling^50^. Our crust is associated with a buoyancy anomaly down to a depth of 150 km. This may exaggerate the effect of a buoyant crust, as the transition to eclogite (that increases the density of the crustal material and cancels the aforementioned buoyancy effect at larger depths) is not included in our model. Therefore we have also tested models where the buoyancy anomaly of the crust was switched off at a depth of 60 km. The results are very similar to models M1 and M2, which suggests that the buoyant crust is essential at shallow depths only – the buoyant shallow part of the crust is sufficient to result in a low dip angle of the slab.

We have shown that the seismicity under the Japan Sea is consistent with the model of subduction of an ageing Pacific slab. Stress fields in the slab are a result of thermal buoyancy of the slab and petrological buoyancy due to the major phase transitions, as well as viscous resistance of the lower mantle. We suggest that seismicity is confined in the upper layer of the subducting slab at shallower depths and in its lower portion at larger depths. These two areas are separated by a stress minimum at ~450 km depth where the stress regime changes from bending to unbending. Lower density of hypocentres as is observed in our studied region, or even the lack of seismicity at 300–500 km depth as observed more to the south at ~40°N, may arise from such a stress minimum, although a contribution from complex 3D structures cannot be ruled out. Though our 2D model is certainly too simplified to fully capture such complicated 3D nature of the considered subduction area, we believe that we identified the primary controls on the stress distribution in the bending slab in the transition zone.

## Supplementary information

Supplementary Information.

## Data Availability

We used seismic locations and focal mechanisms retrieved from the USGS and GCMT public repositories, respectively.
