# Supplementary material for: Geodynamic subduction models constrained by deep earthquakes beneath the Japan Sea and eastern China
Source: Sci Rep. 2020 Mar 25;10:5440. doi: 10.1038/s41598-020-62238-x (PMC7096453; doi:10.1038/s41598-020-62238-x)
Supplement: Supplementary file 1 — Supplementary Information. [file 41598_2020_62238_MOESM1_ESM.pdf]

Supplementary information of the manuscript

Geodynamic subduction models constrained by deep earthquakes beneath the Japan Sea and eastern China

by

Hana Čížková[1*], Jiří Zahradník[1], Junqing Liu[2], Craig R. Bina[3]

[1] Charles University, Faculty of Mathematics and Physics, Department of Geophysics, Prague, Czech Republic

[2] Jilin Earthquake Agency, China Earthquake Administration, Changchun, China

[3] Northwestern University, Department of Earth and Planetary Sciences, Evanston, IL, U.S.A.

Rheology:

We use composite rheological model [1] that combines diffusion creep, dislocation creep, and a power-law stress-limiter [2]. The effective viscosity is calculated from the viscosities of individual creep mechanisms as follows:

$$\eta_{eff} = \left(\frac{1}{\eta_{diff}} + \frac{1}{\eta_{disl}} + \frac{1}{\eta_y}\right)^{-1}. \qquad (1)$$

Here η_{diff}, η_{disl}, and η_y are viscosities of diffusion creep, dislocation creep, and stress-limiter respectively:

$$\eta_{diff} = \frac{1}{A_{diff}} \exp\left(\frac{E_{\mathrm{diff}} + pV_{diff}}{RT}\right), \qquad (2)$$

$$\eta_{disl} = \frac{1}{A_{disl}^{\frac{1}{n}}} e^{(1-n)/n} \exp\left(\frac{E_{disl} + pV_{disl}}{nRT}\right), \qquad (3)$$

$$\eta_y = \tau_y e_y^{-1/n_y} e^{1/n_y - 1}. \qquad (4)$$

In the upper mantle we assume activation parameters based on dry olivine rheology [3]. Only diffusion creep is applied in the lower mantle, with activation parameters based on slab sinking speed analysis [4]. For meanings of symbols and parameter values, see Supplementary Table 1.

Thermal expansivity:

Thermal expansivity decreases with depth [5,6]. We apply the formula [7,8]:

$$\alpha = \alpha_0 \frac{\Delta\alpha}{\left[\left(\Delta\alpha^{1/3} - 1\right)\left(1 - z\right) + 1\right]^3} . \qquad (5)$$

Surface value α_0 and contrast over the mantle $\Delta\alpha$ are given in Supplementary Table 1; *z* is dimensionless depth.

Supplementary Table 1 - Symbols and model parameters

Symbol	Meaning	Value	Units
**Upper mantle and transition zone rheology**			
A_{diff}	Pre-exponential parameter of diffusion creep	1×10^{-9}	$Pa^{-1}\ s^{-1}$
A_{disl}	Pre-exponential parameter of dislocation creep	3.1×10^{-17}	$Pa^{-n}\ s^{-1}$
E_{diff}	Activation energy of diffusion creep	3.35×10^{5}	$J\ mol^{-1}$
E_{disl}	Activation energy of dislocation creep	4.8×10^{5}	$J\ mol^{-1}$
V_{diff}	Activation volume of diffusion creep	4.0×10^{-6}	$m^3\ mol^{-1}$
V_{disl}	Activation volume of dislocation creep	11×10^{-6}	$m^3\ mol^{-1}$
n	Power-law exponent	3.5	-
η_{crust}	Viscosity of crust	10^{20}	Pa s
τ_y	Yield stress	5×10^{8}	Pa
e_y	Reference strainrate	10^{-15}	s^{-1}
n_y	Stress limiter exponent	10	-
p	Hydrostatic pressure	-	Pa
R	Gas constant	8.314	$J\ K^{-1}\ mol^{-1}$
T	Temperature	-	K
e	Second invariant of the strainrate	-	s^{-1}
**Lower mantle rheology**			
A_{diff}	Pre-exponential parameter of diffusion creep	1.3×10^{-16}	$Pa^{-1}\ s^{-1}$
E_{diff}	Activation energy of diffusion creep	2×10^{5}	$J\ mol^{-1}$
V_{diff}	Activation volume of diffusion creep	1.1×10^{-6}	$m^3\ mol^{-1}$
**Other model parameters**			
κ	Diffusivity	10^{-6}	$m^2\ s^{-1}$
G	Gravitational acceleration	9.8	$m\ s^{-2}$
ρ_0	Reference density	3416	$kg\ m^{-3}$
c_p	Specific heat	1250	$J\ kg^{-1}\ K^{-1}$
α_0	Surface thermal expansivity	3×10^{-5}	K^{-1}
$\Delta\alpha$	Expansivity contrast	0.415	-
γ_{410}	Clapeyron slope 410 km phase transition	1×10^{6}, 2×10^{6}	$Pa\ K^{-1}$
γ_{660}	Clapeyron slope 660 km phase transition	-1.5×10^{6}, -2.5×10^{6}	$Pa\ K^{-1}$
$\delta\rho_{410}$	Density contrast 410 km phase transition	273	$kg\ m^{-3}$
$\delta\rho_{660}$	Density contrast 660 km phase transition	341	$kg\ m^{-3}$

References

1. van den Berg, A. P., van den Keken, P. E. & Yuen, D. A. The effects of a composite non-Newtonian and Newtonian rheology on mantle convection. *Geophys. J. Int.* **115**, 62–78 (1993).
2. van Hunen, J., van den Berg, A. P. & Vlaar, N. J. On the role of subducting oceanic plateaus in the development of shallow flat subduction. *Tectonophysics* **352**, 317–333 (2002).
3. Hirth, G. & Kohlstedt, D. Rheology of the upper mantle and mantle wedge: A view from the experimentalists. *In Inside the Subduction Factory. Geophysical Monograph 138, American Geophysical Union, Washintgon, DC.* (2003).
4. Čížková, H., van den Berg, A. P., Spakman, W. & Matyska, C. The viscosity of Earth's lower mantle inferred from sinking speed of subducted lithosphere. *Phys. Earth Planet. Inter.* **200**–**201**, 56–62 (2012).
5. Chopelas, A. & Boehler, R. Thermal expansivity in the lower mantle. *Geophys. Res. Lett.* **19**, 1983–1986 (1992).
6. Katsura, T. *et al.* P-V-T relations of $MgSiO_3$ perovskite determined by in situ X-ray diffraction using a large-volume high-pressure apparatus. *Geophys. Res. Lett.* **36**, L01305 (2009).
7. Hansen, U. & Yuen, D. A. Effects of depth-dependent thermal expansivity on the interaction of thermal-chemical plumes with a compositional boundary. *Phys. Earth Planet. Inter.* **86**, 205–221 (1994).
8. Steinbach, V. & Yuen, D. A. Effects of depth-dependent properties on the thermal anomalies produced in flush instabilities from phase transitions. *Phys. Earth Planet. Inter.* **86**, 165–183 (1994).
